# Sutureless technique combined with vertical vein incision and pulmonary veins unroofed for correction of infracardiac total anomalous pulmonary venous connection

**DOI:** 10.3389/fped.2023.1180040

**Published:** 2023-07-10

**Authors:** Zhen Bing, Rui Chen, Quansheng Xing, Pengchao Xing, Bei Lv

**Affiliations:** Heart Center, Qingdao Women and Children's Hospital, Affiliated to Qingdao University, Qingdao, China

**Keywords:** total anomalous pulmonary venous connection, infracardiac, sutureless technique, vertical vein, total anomalous pulmonary venous connection surgery, semisutureless technique in infracardiac TAPVC surgery

## Abstract

**Objective:**

We report a surgical method (sutureless technique), combined with vertical vein incision and pulmonary veins unroofed (semisutureless technique), to correct infracardiac total anomalous pulmonary venous connection (TAPVC).

**Materials and methods:**

The clinical characteristics of 21 patients, who were diagnosed with infracardiac TAPVS between February 2017 and March 2022, were retrospectively analyzed. These patients were divided into three groups according to different surgical methods: conventional surgery group, sutureless technique group, and semisutureless technique group. The conventional surgery group enrolled five patients with a median age of 16 days (interquartile range, 9–27 days) and a median weight of 3.25 kg (interquartile range, 3.1–3.42 kg). In this group, no preoperative pulmonary vein obstruction (PVO), preoperative ventilator support, or emergency surgery were reported. The sutureless technique group enrolled seven patients with a median age of 12 days (interquartile range, 5–16 days) and a median weight of 3.04 kg (interquartile range, 2.76–3.20 kg). In this group, two patients with preoperative PVO, four patients with preoperative ventilator support, and seven patients requiring emergency operation were found. The semisutureless technique group enrolled nine patients with a median age of 14 days (interquartile range, 7–24 days) and a median weight of 3.22 kg (interquartile range, 3.15–3.50 kg). In this group, four patients with preoperative PVO, two patients with preoperative ventilator support, and seven patients requiring emergency operation were noted.

**Results:**

In the conventional surgery group, two patients with postoperative supraventricular tachycardia, one patient with postoperative low cardiac output syndrome, one patient with PVO, and no case of postoperative death were reported. In the sutureless technique group, two patients with postoperative low cardiac output syndrome, one patient with postoperative supraventricular tachycardia, one patient with postoperative PVO, and no postoperative deaths were determined. In the semisutureless technique group, three patients had low cardiac output syndrome, two patients had supraventricular tachycardia after the operation, and one patient, who had been admitted to the hospital after cardiopulmonary resuscitation in the emergency room, died early after the operation. No case of death or PVO was noted after the operation.

**Conclusion:**

The semisutureless technique has positive effects. This surgery method can enlarge the anastomotic stoma, increase the volume of the left atrium, reduce the tension of the anastomotic stoma, fix the pulmonary vein to avoid distortion, and prevent postoperative hemorrhage.

## 1. Introduction

Total anomalous pulmonary venous connection (TAPVC) is a rare complex congenital heart disease (CHD). According to Darling's classification, TAPVC can be divided into four types: the supracardiac type, the intracardiac type, the infracardiac type, and the mixed type (1). The infracardiac TAPVC accounts for approximately 20%–30% of all cases. The pathophysiology and clinical manifestation of infracardiac TAPVC is different from other subtypes, and often early surgery is recommended immediately upon diagnosis. The surgical method includes conventional surgery and sutureless technique. The conventional surgery is to anastomose the pulmonary veins with the left atrium (LA). The sutureless technique is to anastomose the posterior pericardium with the LA. Compared with the conventional surgery, the sutureless technique can help avoid damaging the pulmonary vein wall, reduce irritation to the pulmonary vein intima, prevent postoperative pulmonary vein intima hyperplasia, and reduce the incidence of postoperative pulmonary vein obstruction (PVO) (2–4). Therefore, the sutureless technique is recommended for patients with infracardiac TAPVC or other types of TAPVC with evidence of PVO (5). However, some limitations to performing the sutureless technique also exist. Potential complications such as anastomotic bleeding, phrenic nerve injury, and air embolism may occur (2). In order to reduce the risk of anastomotic bleeding, expand the anastomosis, and reduce the tension of the anastomosis, we used the sutureless technique, combined with vertical vein incision and pulmonary veins unroofed (semisutureless technique), to correct infracardiac TAPVC.

## 2. Materials and methods

### 2.1. Clinical data of patients

**Figure 1:**
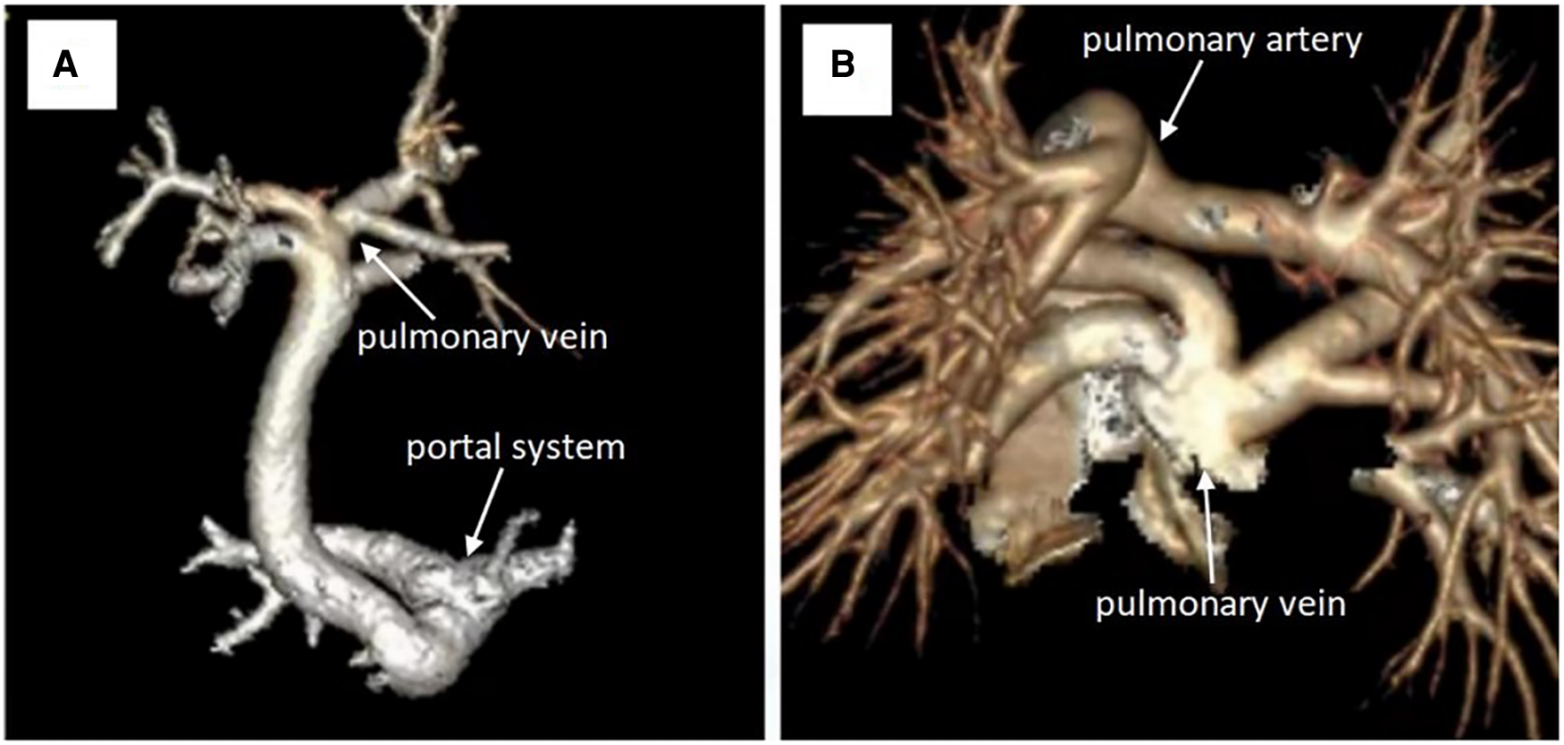
(**A**) CT angiography reconstruction for the preoperative pulmonary vein. (**B**) CT angiography reconstruction for the postoperative pulmonary vein .

A total of 21 consecutive patients with infracardiac TAPVC who underwent conventional repair (CR) (*n* = 5), sutureless repair (*n* = 7), or semisutureless repair (*n* = 9) between 1 February 2017 and 31 March 2022 were included. They were divided into three groups according to the surgical methods. First, five patients were enrolled in the traditional surgery group, consisting of three males and two females, with a median age of 16 days (interquartile range, 9–27 days) and a median weight of 3.25 kg (interquartile range, 3.1–3.42 kg). All five children had atrial septal defect (ASD) or patent foramen oval (PFO), and two of them had patent ductus arteriosus (PDA). In this group, no patient with preoperative PVO, preoperative ventilator support, or requiring emergency surgery were noted. Second, seven patients were enrolled in the sutureless repair group, which consisted of three males and four females, with a median age of 12 days (interquartile range, 5–16 days) and a median weight of 3.04 kg (interquartile range, 2.76–3.20 kg). All seven children had ASD or PFO, and four of them had PDA. Two patients with preoperative PVO, four patients with preoperative ventilator support, and seven patients requiring emergency operation were reported. Third, nine patients were enrolled in the semi-sutureless technique repair group, which consisted of five males and four females, with a median age of 14 days (interquartile range, 7–24 days) and a median weight of 3.22 kg (interquartile range, 3.15–3.50 kg). All nine children had ASD or PFO, and six of them had PDA. Four patients with preoperative PVO, two patients with preoperative ventilator support, and seven patients requiring emergency operation were identified (Table 1). Echocardiography, chest radiograph, and electrocardiogram were performed on all patients for preoperative evaluation. A total of 17 patients preoperatively underwent cardiac computed tomography angiography (CTA) (Figure 1). Four patients were hemodynamically unstable and underwent emergency operation without prior CTA.

**Table 1:** Clinical data of 21 patients with infracardiac TAPVC.

Clinical data	Conventional	Sutureless	Semisutureless
Number of cases	5	7	9
Median age in days (IQR)	16 (9–27)	12 (5–16)	14 (7–24)
Median weight in kg (IQR)	3.25 (3.1–3.42)	3.04 (2.76–3.20)	3.22 (3.15–3.50)
Combined malformation (number of cases, disease)	5 ASD or PFO 2 PDA	7 ASD or PFO 4 PDA	9 ASD or PFO 6 PDA
Preoperative PVO	0	2	2
Ventilator support before operation	1	2	4
Emergency operation	0	4	7
Median CPB in minutes (IQR)	96.5 (60–111)	85.5 (55–125)	79 (52–108)
Median aortic cross-clamp in minutes (IQR)	57 (36–73)	46 (28–58)	43 (25–61)
Delayed sternal closure	2	1	2
Median ICU stay time in hours (IQR)	104 (78–189)	98 (72–168)	94 (68–172)
Median postoperative ventilator time in hours (IQR)	98 (65–143.5)	87 (56–139)	93.5 (61–141)
Postoperative LCOS	1	2	3
Postoperative supraventricular tachycardia	2	1	2
Early postoperative death	0	0	1
Intermediate postoperative death	0	0	0
Postoperative PVO	1	1	0
Missed follow-up	0	1	0

TAPVC, total anomalous pulmonary venous connection; PVO, pulmonary vein obstruction; LCOS, low cardiac output syndrome; CPB, cardiopulmonary bypass.

### 2.2. Surgical method

**Figure 2:**
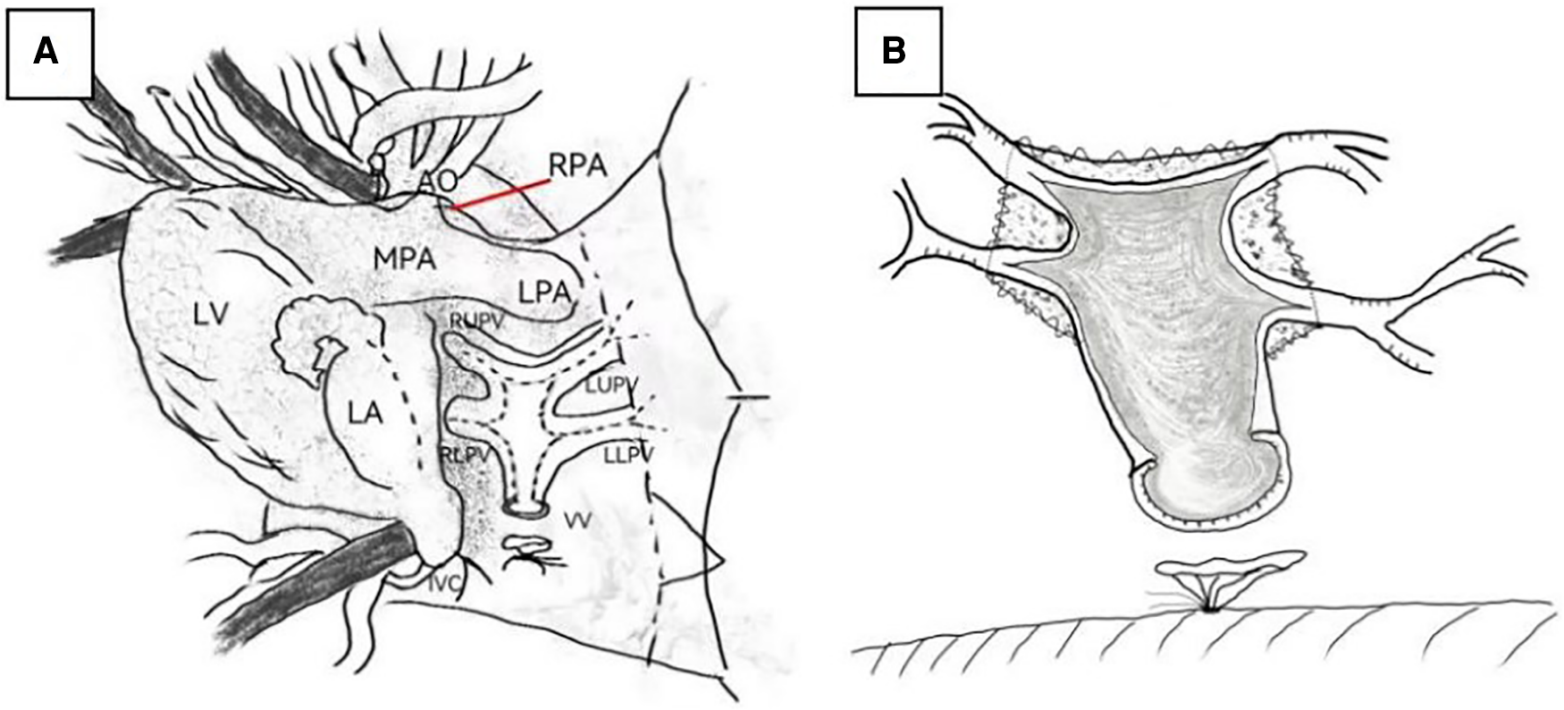
(**A**) The incision line of the left atrium, vertical vein, and pulmonary vein. (**B**) The incision and unroofed pulmonary veins. LA, left atrium; LV, left ventricle; MPA, main pulmonary artery; LPA, left pulmonary artery; RPA, right pulmonary artery; AO, aorta; LLPV, left lower pulmonary vein; LUPV, left upper pulmonary vein; RLPV, right lower pulmonary vein; RUPV, right upper pulmonary vein; VV: vertical vein.

The conventional surgery and sutureless technique for TAPVC are advanced, and, therefore, we will not repeat them here. We will focus on the surgical process of the semisutureless technique. The repair was performed through a mid-line sternotomy. The PDA was ligated before the commencement of cardiopulmonary bypass (CPB) if it existed. The conventional aorta and superior and inferior vena cava were cannulated to establish CPB. Systemic cooling to a rectal temperature of 25°C was performed, while the aorta was cross-clamped, and electromechanical arrest was accomplished by delivering cold blood cardioplegic solution to the aortic root. The apex of the heart was turned to the right chest, and the common pulmonary vein was exposed through the oblique pericardial sinus. The vertical vein was ligated at the level of the diaphragm. The left atrial incision was performed along the long axis (Figure 2A). The left atrial wall and the pericardium were anastomosed without incision of the pulmonary and vertical veins. After two-thirds of the anastomosis was completed, the vertical vein was transected and longitudinally opened. Subsequently, the pulmonary veins were cut and unroofed (Figure 2A). Being unroofed means that the anterior walls of the vertical vein and pulmonary vein were partly excised (Figure 2B), and the remaining third of the anastomosis was completed between the LA and the vertical vein. At the same time, ASD was repaired.

### 2.3. Observation indicators

The endpoint was defined as early, intermediate, or late mortality or PVO. Early was defined as death up to 30 days from the day of surgery, while intermediate was up to 90 days, and late was beyond 90 days after surgery. PVO had an echocardiographic Doppler velocity of 1.8 m/s or more, a catheterization gradient of 4 mmHg or more, or 50% or more narrowing by angiography (6). Emergency operation was defined as a lifesaving surgery performed within 24 h of presentation. Selective operation was defined as surgery performed beyond 24 h after admission.

### 2.4. Statistical methods

Continuous data are presented as mean standard deviation or as median [interquartile range (IQR)] according to data distribution. Categorical data are presented as absolute numbers and percentages. All analyses were performed by using SPSS software version 25.0. Due to the small sample size and non-randomization, only statistical descriptions are provided without comparing the differences between the groups.

## 3. Results

The conventional surgery group had a median CPB time of 96.5 min (interquartile range, 60–111 min) and a median aortic cross-clamp time of 57 min (interquartile range, 37–73 min). In this group, two patients with delayed sternal closure were found. The median ICU stay time was 104 h (interquartile range, 78–189 h), and the median postoperative ventilator time was 98 min (interquartile range, 65–143.5 min). One patient with postoperative low cardiac output syndrome (LCOS), two patients with postoperative supraventricular tachycardia, and no case of postoperative death were identified. A case of a patient with PVO was found 5 months after the operation. The echocardiogram showed that the flow velocity of the right upper pulmonary vein was 1.9 m/s. No mortality was reported, and no patient was lost to follow-up (Table 1).

The sutureless technique group had a median CPB time of 79 min (interquartile range, 52–108 min) and a median aortic cross-clamp time of 46 min (interquartile range, 28–108 min). Only one patient with delayed sternal closure was found. The median ICU stay time was 98 h (interquartile range, 72–168 h), and the median postoperative ventilator time was 87 min (interquartile range, 56–139 min). Two patients with postoperative LCOS, one patient with postoperative supraventricular tachycardia, and no case of postoperative death were found. In the follow-up of up to 38 months, one patient developed PVO. This patient had preoperative PVO. However, the patient's condition is now stable, and the follow-up will continue. The echocardiography showed that the left upper pulmonary vein was 2.2 m/s, the right lower pulmonary vein was 2.1 m/s, and the pulmonary artery systolic pressure was 45 mmHg. The CTA showed that the PVO was not severe and surveillance was ongoing. One patient was lost to follow-up (Table 1).

The semisutureless technique group had a median CPB time of 85.5 min (interquartile range, 55–125 min) and a median aortic cross-clamp time of 43 min (interquartile range, 25–61 min). Delayed sternal closure was needed in two patients. The median ICU stay time was 94 h (interquartile range, 68–172 h), and the median postoperative ventilator time was 93.5 min (interquartile range, 61–141 min). Three patients with postoperative LCOS and two patients with postoperative supraventricular tachycardia were found. One patient died early after the operation. They were admitted to the hospital after cardiopulmonary resuscitation in the emergency room. During the emergency operation, the left upper, left lower, and right lower pulmonary veins were found to have dysplasia. No intermediate mortality, no incidence of PVO, and one mortality for this group were determined. No patient was lost to follow-up (Table 1).

## 4. Discussion

The vertical veins of infracardiac TAPVC run far and pass through the diaphragm. It is easily affected by the activities of breathing, crying, and other factors. In addition, the portal vein system has high resistance, which will have a certain impact on the blood flow of the pulmonary vein. Most patients with infracardiac TAPVC have symptoms of PVO in the neonatal period (7). PVO can be manifested as pulmonary congestion and pulmonary hypertension, as well as decreased systemic blood flow and severe hypoxia, resulting in systemic hypoperfusion and metabolic acidosis. It often requires emergency or subemergency surgical treatment in the neonatal period. Due to the small infant age, low weight, and the development of pulmonary veins, the mortality rate after surgery in the neonatal period is high, and its surgical treatment involves great challenges (2).

The infracardiac TAPVC is an independent risk factor for postoperative death and PVO (8, 9). In order to improve the prognosis of infracardiac TAPVC, cardiac surgery experts have been making attempts to improve the surgical scheme, including the improvement of the surgical path, the design of an anastomotic shape, and the application of the sutureless technique (10). The sutureless technique can avoid mechanical stimuli to PVs and minimize suture line distortion arising from complex geometry, thereby decreasing postrepair PVO (11). A systematic review and meta-analysis showed that, compared with conventional surgical methods for the treatment of TAPVC, the sutureless technique significantly reduced early mortality, total mortality, postoperative PVO, and reoperation risk by 53%, 45%, 77%, and 67%, respectively (3). The principle of the sutureless technique is controlled hemorrhage into the pericardial cavity and then redirecting this into the LA. The disadvantage is a requirement of a meticulous technique to ensure that hemorrhage is contained within the LA, and if the incisions on PVs extend into the pleural cavity, the bleeding may be difficult to diagnose or control (11, 12).

In this study, nine patients underwent surgery with the semisutureless technique. They had hypoplasia of the pulmonary vein or decreased left atrial volume. In this group, only one patient died in the early postoperative period. He was admitted to the hospital after cardiopulmonary resuscitation in the emergency room. During the emergency operation, the left upper, left lower, and right lower pulmonary veins were found to be diffusely hypoplastic to the hilar lung parenchyma, where the normal-looking vein could not be seen. The effect of surgery on the semisutureless technique group was generally satisfactory. In addition to avoiding injury and stimulation of pulmonary veins to cause postoperative scar hyperplasia at the anastomosis, it also has the following five advantages: (1) It can postoperatively prevent the hemorrhage of pulmonary vein confluence and pericardium for making full use of the vertical vein tissue and reducing the tension of the anastomosis. (2) It can maximize the anastomosis with the LA and reduce the probability of postoperative restenosis to avoid the distortion and stenosis of anastomosis caused by high tension. (3) It can increase the left atrial volume. (4) Suture of the vertical vein to the LA wall plays a certain role in fixing the pulmonary vein and preventing the distortion of the pulmonary vein after the heart beats and (5) It is beneficial to reduce hemorrhage of the pulmonary veins during operation, keep the operation field clear, and shorten the time of the circulatory arrest. The anastomosis of the left atrial wall and the pericardium was a closed chamber anastomosis without incision of the pulmonary vein and the vertical vein. After two-thirds of the anastomosis was completed, the vertical and pulmonary veins were cut and unroofed, and the remaining third of the anastomosis was completed, that is, the anastomosis of the vertical vein and the left atrial wall.

This study has some limitations. Due to the small number of cases and non-random groupings, the preoperative and postoperative data cannot be statistically compared.

In conclusion, the correction effect of TAPVC operation in our heart center is satisfactory. The sutureless technique, combined with vertical vein incision and pulmonary veins unroofed (semisutureless technique), can be used for the correction of infracardiac TAPVC. It is especially suitable for children with pulmonary venous dysplasia or small left atrial volume. It can expand the anastomosis, increase the left atrial volume, reduce the tension of the anastomosis, fix the pulmonary vein to avoid distortion, and prevent postoperative common vein and pericardial hemorrhage. However, more follow-up clinical practice and verification are needed.

## Data Availability

The original contributions presented in the study are included in the article, and further inquiries can be directed to the corresponding author.
